# Comparison of the safety and efficacy of radiofrequency thermocoagulation with percutaneous balloon compression for treating trigeminal neuralgia: a systematic review and meta-analysis

**DOI:** 10.3389/fneur.2023.1178335

**Published:** 2023-09-06

**Authors:** Zeyu Wu, Yongming Zhao, Jiang Liu, Yiyue Fan, Ying Yang

**Affiliations:** ^1^Department of Pain Management, The Affiliated Nanchong Central Hospital of North Sichuan Medical College, Nanchong, Sichuan, China; ^2^The Affiliated Hospital of North Sichuan Medical College, Nanchong, Sichuan, China

**Keywords:** trigeminal neuralgia, radiofrequency thermocoagulation, percutaneous balloon compression, curative effect, meta-analysis

## Abstract

**Objective:**

This study aimed to systematically assess the efficacy and complications of radiofrequency thermocoagulation (RFT) and percutaneous balloon compression (PBC) for treating trigeminal neuralgia (TN).

**Methods:**

Chinese and English studies on RFT and PBC in the treatment of TN were systematically searched using CNKI, Wanfang Data, VIP, PubMed, EMBASE, Cochrane Library, and until December 31, 2022. Further, the literature was strictly screened using specific inclusion and exclusion criteria. The RevMan 5.4 software was used for data processing and meta-analysis.

**Results:**

Overall, 16 studies with 3,326 patients were included. The results of meta-analysis revealed that no significant difference was present between the two groups in terms of the rate of efficacy immediately after surgery, 1 month after surgery, and 3 months after surgery (odds ratio [OR] = 0.73, 95% confidence interval [CI] 0.35–1.54, *p* = 0.41; OR = 0.41, 95% CI 0.13–1.32, *p* = 0.13; OR = 0.40, 95% CI 0.10–1.60, *p* = 0.20); however, at 12 months after surgery, the difference was statistically significant (OR = 0.27, 95% CI 0.10–0.75, *p* = 0.01). Notably, there was no significant difference in the postoperative sleep quality index between the two groups immediately after surgery and 1 month after surgery (*SMD* = −0.01, 95% CI −2.47 to 2.44, *p* = 0.99; *SMD* = 0.14, 95% CI −3.95 to 4.22, *p* = 0.95). Further, statistically significant differences were observed between the two groups in the incidence of postoperative masticatory muscle strength decline and oral herpes (OR = 0.37; 95% CI 0.21–0.63, *p* = 0.0003; OR = 0.25, 95% CI 0.10–0.61, *p* = 0.003). In addition, a statistically significant difference was found in the recurrence rate at 1-year follow-up (OR = 2.23, 95% CI 1.03–4.81, *p* = 0.04); however, no statistically significant differences were found in the recurrence rate at the 2-year follow-up (OR = 1.95, 95% CI 0.33–11.59, *p* = 0.46).

**Conclusion:**

In the treatment of TN, both RFT and PBC can achieve good short-term efficacy, and no significant differences were noted between the outcomes of the two approaches. Compared with RFT, PBC may result in a lower pain score and recurrence rate in the medium and long terms, but it is a higher incidence of cold sores, and the decrease of masticatory muscle strength is more obvious.

## 1. Introduction

Trigeminal neuralgia (TN) refers to chronic neuropathic pain that manifests as spontaneous, electric shock-like, or pins-and-needles-type paroxysmal pain on the face. It is recognized as a common cause of maxillofacial headache (1). Further, depending on the cause, TN can be classified into the following types: classic (primary), secondary, or idiopathic. According to Maarbjerg et al. (2), the etiology of primary TN is currently unknown, but it may be attributed to ectopic pulses and tactile crosstalk caused by the demyelination of primary sensory neurons of the afferent trigeminal nerve. Sahoo and Perez (3, 4) reported that vascular compression leading to microvascular injury initiating focal demyelination may be linked to local changes that lower the excitability threshold of affected nerve fibers and facilitate inappropriate synaptic propagation to adjacent fibers. Further, Mannerak et al. (5) reported that the occurrence of TN may be linked to genetic factors. According to Lambru et al. (6), the lifetime prevalence of TN ranges from 0.16 to 0.3%, with an annual incidence ranging from 4 to 29/100,000 person years. The incidence of TN increases with increasing age, and the average age of onset in adults is 53–57 years. Carbamazepine—an antiepileptic drug—is the drug of choice for the treatment of TN and can provide satisfactory results. Moreover, this drug can block voltage-gated sodium channels, stabilizing the overexcited neuronal membrane and inhibiting competitive firing (7, 8). Liu Chong et al. (9) introduced surgical treatment for TN, which included microvascular decompression and radiofrequency thermocoagulation (RFT). Furthermore, percutaneous balloon compression (PBC), stereotactic radiotherapy, and RFT are the most widely used treatment options for TN. The efficacy of RFT and PBC in the treatment of TN is debatable. For example, according to Zhang Jian and Yu Liang (10, 11), RFT is more effective than PBC in the immediate postoperative period. Conversely, according to Chen Yanru, Maiira Mamaidi, and Meglio et al. (12–14), PBC is more effective than RFT in the immediate postoperative period. However, to the best of our knowledge, there are no large-sample multicenter studies comparing the efficacy of the two procedures. Thus, this study aimed to propose evidence-based clinical treatment by systematically evaluating the effectiveness, recurrence, and complications of RFT and PMC in the treatment of TN.

## 2. Materials and methods

This systematic review and meta-analysis was conducted in accordance with the Preferred Reporting Items for Systematic reviews and Meta-Analyses guidelines (CRD42023387069).

### 2.1. Search strategy and selection criteria

The search was conducted by two independent investigators (Wu, Fan). Any disagreements or discrepancies were resolved by consensus. Computer search of CNKI, Wanfang data, VIP, PubMed, EMbase, CochraneLibrary and other Chinese and English database, The time is from the establishment of the database to December 31, 2022.Chinese search terms: “trigeminal neuralgia,” “radiofrequency thermocoagulation,” “microballoon compression” and their respective synonyms and synonyms. English search words: ((((((((((Radiofrequency Ablation) OR (Ablation, Radiofrequency)) OR (Radio Frequency Ablation)) OR (Ablation, Radio Frequency)) OR (Radio-Frequency Ablation)) OR (Ablation, Radio-Frequency)) OR (radiofrequency thermocoagulation)) OR (RF thermocoagulation)) OR (Thermocoagulation)) AND ((((((microballoon compression) OR (balloon compression)) OR (PBC)) OR (PBC)) OR (percutaneous balloon compression)) OR (percutaneous microballoon compression))) AND (((((((((((((((((((((trigeminal neuralgia) OR (Neuralgia, Trigeminal)) OR (Trigeminal Neuralgias)) OR (Tic Doloureux)) OR (Fothergill Disease)) OR (Disease, Fothergill)) OR (Trifacial Neuralgia)) OR (Neuralgia, Trifacial)) OR (Trifacial Neuralgias)) OR (Tic Douloureux)) OR (Epileptiform Neuralgia)) OR (Epileptiform Neuralgias)) OR (Neuralgia, Epileptiform)) OR (Trigeminal Neuralgia, Idiopathic)) OR (Idiopathic Trigeminal Neuralgia)) OR (Idiopathic Trigeminal Neuralgias)) OR (Neuralgia, Idiopathic Trigeminal)).

### 2.2. Inclusion and exclusion criteria

Inclusion criteria were as follows: clinical trials of RFT and PBC in the treatment of TN published before December 31, 2022 in the United States and abroad; studies of patients with a clinical diagnosis of primary TN or secondary recurrence after surgery; studies in which the intervention measures were only RFT and PBC; and studies in which outcome measures included pain relief, postoperative recurrence, and complications.

Exclusion criteria were as follows: studies of patients with secondary TN (tumor and multiple sclerosis); relevant papers without clear data (such as reviews, case reports, and conference papers); papers in which outcome measures did not include effective rate, recurrence rate, or complication rate; studies with unclearly reported follow-up time and results; and studies involving other intervention measures.

### 2.3. Outcome evaluation index

We compared the safety and efficacy of RFT and PBC in the treatment of TN using the following primary outcomes: digitally measured pain intensity scale (NRS) or visual analog scale (VAS) score, rate of efficiency, rate of complications (such as numbness, decreased masticatory muscle strength, cold sores, and corneal discomfort), rate of recurrence, and sleep quality score. If the definition of the results differed, we categorized the authors’ original results according to the specific criteria.

### 2.4. Literature quality evaluation

After thoroughly reading the full text, two researchers with relevant knowledge strictly screened the literature according to the specific inclusion and exclusion criteria and extracted the relevant data. In cases of disagreement, the entire research team discussed and made the decision as a group. The improved Jadad scale was used to assess the quality of randomized controlled trials, with scores ranging from 0 to 7 and scores ranging from 4 to 7 as high quality literature, respectively. To assess the quality of case–control and cohort studies, the Newcastle–Ottawa scale (NOS) was used, with the total score was 10 and the scores of ≥5 indicating high-quality literature.

### 2.5. Statistical methods

The RevMan 5.4 software was used for data processing and analysis. The M–H method was used for statistical analysis. When the *p*-value was >0.05, *I*^2^ of <50% was used for statistical analysis; otherwise, random effect model analysis was selected, and subgroup or sensitivity analysis was performed to determine the source of heterogeneity. The effect size was calculated using the odds ratio (OR), and the judgment index was calculated using 95% confidence interval (CI) of the effect size. A *p*-value of <0.05 was considered statistically significant.

## 3. Results

### 3.1. Literature screening results

**Figure 1:**
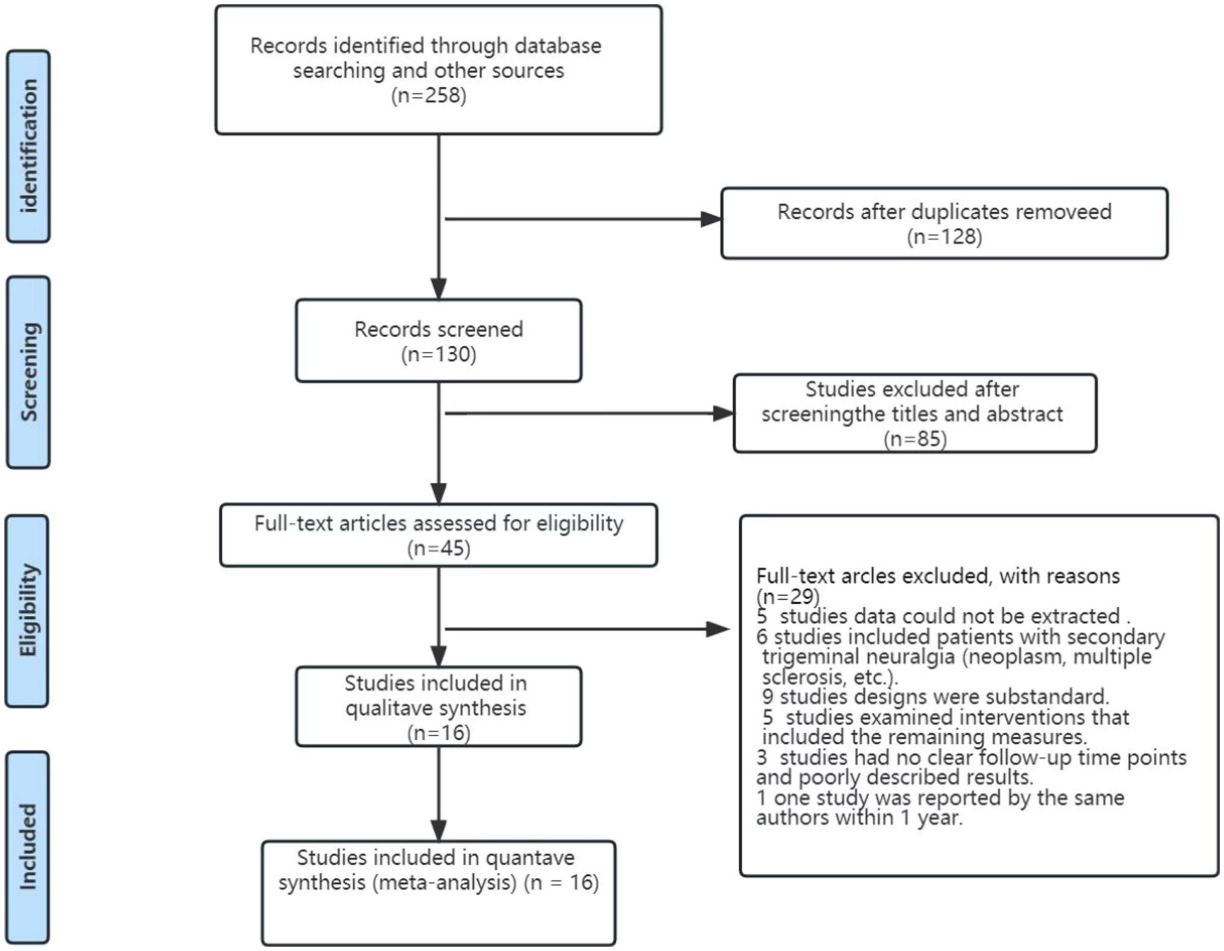
PRISMA flow chart for study identification and inclusion/exclusion.

Initially, 258 studies were examined. The Endnote X9 software was used to resend these studies, after which the titles and abstracts were screened before reading the full text. Finally, 16 studies with 3,326 patients were included—12 in Chinese and 4 in English (Figure 1).

The RFT and PBC groups included 1,966 and 1,360 cases, respectively. The following data were extracted: first author, publication year, patient age, onset course, onset location, pain score, operation, and outcome index. Overall, 12 observational studies had the NOS scores of ≥5. Further, four randomized controlled trials had the Jadad scores of ≥4, and all of them were of high quality (Table 1).

**Table 1:** Basic characteristics of the included literature for RFT versus PBC for TN.

Study	Sample size	Sex (M/F)	Age (y, Mean ± SD)	Course of disease (y, Mean ± SD)	Affected side(L/R)	Pain range (V1, V2, V3)	Pre-operative score (VAS/NRS)	Treatment	Outcomes	Time of follow-up(y)	The type of research	Quality score (NOS, Jadad)
Chen et al. (12)	P:30 R:30	P:10/20 R:12/18	P:66 ± 9 R:67 ± 8	P:2.5 ± 2.4 R:3.3 ± 3.3	P:10/20 R:11/19	P:V2(9), V2 + V3(13), V3(8) R:V2(11), V2 + V3(15), V3(4)	P:6.17 ± 1.02 R:6.53 ± 1.07	P:oppress 5 mins R: 75–80°C	1. NRS 2. BNI 3. Effective rate 4. Recurrence rate 5. Adverse event	1. Immediately after surgery 2. 1 week 3. 1 months 4. 3 months 5. 6 months 6. 12 months	Retrospective study	8
Wang et al. (15)	P:40 R:40	P:15/25 R:17/23	P:63.32 ± 5.31 R:59.63 ± 3.79	P:8.35 ± 1.63 R:7.56 ± 2.57	P:16/24 R:18/22	None	P:8.51 ± 0.78 R:8.36 ± 0.69	P:oppress 2.5–3.5 mins R:40–75°C(RF for 1 min for every 5°C degrees,75°C 2 mins)	1. NRS 2. PSQI 3. Effective rate 4. Recurrence rate 5. Adverse event 6. Inflammatory factor levels	1. 3 days 2. 1 month 3. 3 month	Retrospective study	7
Ding et al. (16)	P:20 R:26	P:9/11 R:12/16	P:67.6 ± 14.19 R:65.3 ± 12.61	None	None	P:V1(1), V1 + V2(8), V1 + V2 + V3(11) R:V1(1), V1 + V2(13), V1 + V2 + V3(12)	P:6.85 ± 0.59 R:6.96 ± 0.66	P:oppress 2-3 mins R: –75°C (2–3 mins, V1<65°C)	1. NRS 2. Adverse event	1. 3 days 2. 3 months	Retrospective study	7
Zhang et al. (17)	P:48 R:49	P:20/28 R:18/31	P:57.6 ± 4.4 R:57.7 ± 4.4	P:1.54 ± 0.42 R:1.61 ± 0.39	None	P:V2(8), V2 + V3(13), V3(27) R:V2(5), V2 + V3(15), V3(29)	P:7.64 ± 1.38 R:7.66 ± 1.41	P:oppress 5 mins R:70–75°C	1. VAS 2. PSQI 3. Effective rate 4. Recurrence rate 5. Adverse event 6. SF-36	1. 1 days 2. 1 week 3. 1mo	Retrospective study	8
Zhang et al. (18)	P:56 R:48	P:23/33 R:23/25	P:68.38 ± 12.65 R:65.18 ± 11.27	P:3(0.81,6) R:4 (2,9)	P:19/37 R:18/30	P:V2(15), V3(10), V1 + V2(7), V1 + V3(0), V2 + V3(23), V1 + V2 + V3(1) R:V2(11), V3(20), V1 + V2(3), V1 + V3(1), V2 + V3(12), V1 + V2 + V3(1)	P:7.43 ± 1.64 R:7.88 ± 1.35	P:oppress 2–3 mins R:60–85°C (1.5–2 mins)	1. VAS 2. BNI 3. Effective rate 4. Recurrence rate 5. Adverse event	1. 1 month 2. 3 months 3. 6 months 4. 12 months	Retrospective study	8
Rong et al. (19)	P:30 R:30	P:12/18 R:11/19	P:69.5 ± 9.5 R:68.2 ± 8.0	P:1.19 ± 0.33 R:1.05 ± 0.42	P:12/18 R:13/17	P:V1(1), V2(10), V3(13), V1 + V2(2), V2 + V3(4) R:V1(0), V2(11), V3(12), V1 + V2(1), V2 + V3(6)	P:7.54 ± 1.33 R:7.42 ± 1.41	P:oppress 2.5 min R:85°C3minute	1. NRS 2. Effective rate(3 m) 3. Adverse event(1w/3 m)	1. Immediately after surgery 2. 1 week 3. 3 months	RCT	7
Zhao et al. (20)	P:21 R:19	P:10/11 R:9/10	P:67.2 ± 10.0 R:67.2 ± 12.5	P:0.38 ± 0.19 R:0.34 ± 0.19	P:8/13 R:8/11	P:V1(1), V2(4), V3(6), V1 + V2(2), V2 + V3(8) R:V1(1), V2(4), V3(5), V1 + V2(2), V2 + V3(7)	None	P:oppress 3 min R:60/70°C 1 min, 75°C 3 min	1. BNI 2. effective rate 3. QoR 4. adverse event 5. Intraoperative hemodynamics	1. Immediately after surgery 2. 3 days 3. 3 months	RCT	7
Zhang et al. (10)	P:43 R:43	P:17/26 R:15/28	P:64.72 ± 5.51 R:65.23 ± 5.74	P:1.75 ± 0.47 R:1.91 ± 0.55		P:V2(7), V3(25), V2 + V3(11) R:V2(10), V3(23), V2 + V3(10)	P:7.62 ± 1.39 R:7.51 ± 1.44	P:oppress 5 min R:55/60/65/7075°C 50–70s	1. VAS/QS 2. Effective rate 3. Recurrence rate 4. Adverse event 5. SF-36	1. Immediately after surgery 2. 12mo	RCT	7
Liang et al. (11)	P:42 R:45	37/50	75.5 ± 12.8	>0.5	None	None	None	P:oppress 5 min R:75/80°C 1.5 min	1. Effective rate 2. Recurrence rate 3. Adverse event	1. Immediately after surgery 2. 6 months	Retrospective study	6
Yuan et al. (21)	P:18 R:36	P:8/10 R:16/20	P:67 ± 8 R:60 ± 13	P:5.00 ± 0.41 R:8.16 ± 7.67	P:6/12 R:11/25	None	None	P:oppress 2–3 min R:60–70°C 1–1.5 min	1. Effective rate 2. Recurrence rate 3. Adverse event	1. Immediately after surgery 2. 24 months	Retrospective study	6
Maiira Mamaidi et al. (13)	P:28 R:47	P:8/20 R:19/28	P:64.00 ± 13.29 R:66.04 ± 13.83	P:3.85 ± 3.89 R:4.26 ± 3.68	error	None	P:7.32 ± 0.66 R:7.00 ± 1.19	P:oppress 2 min R:60–80°C 1–2 min	1. VAS/QS 2. Effective rate 3. Recurrence rate 4. Adverse event 5. SAS/SDS	1. Immediately after surgery 2. 1 month 3. 6 months	Retrospective study	8
Frank et al. (22)	P:212 R:700	None	None	None	None	None	None	P:oppress 2-3 min R:65–75°C 1.5–2 min	1. Recurrence rate 2. Adverse event	1. 36 months	Retrospective study	5
Meglio et al. (14)	P:74 R:33	None	None	None	None	None	None	P:oppress 1–10 min R:75–80°C 1–3 min	1. Effective rate 2. Recurrence rate 3. Adverse event	1. Immediately after surgery 2. 12 months 3. 24 months 3. 36 months	Retrospective study	6
Zheng et al. (23)	P:664 R:786	P:277/387 R:312/474	None	None	P:250/405/9 R:320/458/8	None	None	P:oppress 3 min R:62, 75°C 1.5 min	1. Adverse event	1. Immediately after surgery	Retrospective study	7
Jain (24)	P:10 R:10	None	None	None	None	None	None	P:oppress 1.5 min R:65–70°C 2 min	1. Recurrence rate 2. Adverse event	1. Immediately after surgery 2. 6 months 3. 12 months 3. 24 months	Retrospective study	7
Wen et al. (25)	P:24 R:24	P:6/18 R:6/18	P:62.33 ± 10.19 R:63.88 ± 11.86	P:8.81 ± 10.16 R:5.76 ± 5.95	P:16/7/2 R:15/8/1	None	P:9 (8.25,10) R:9 (8,10)	P:oppress 5 min R:75°C 2 min	1. VAS 2. Effective rate 3. Recurrence rate 4. Adverse event 5. SAS/SDS	1. Immediately after surgery 2. 12 months	Retrospective study	6

### 3.2. Efficiency comparison

**Figure 2:**
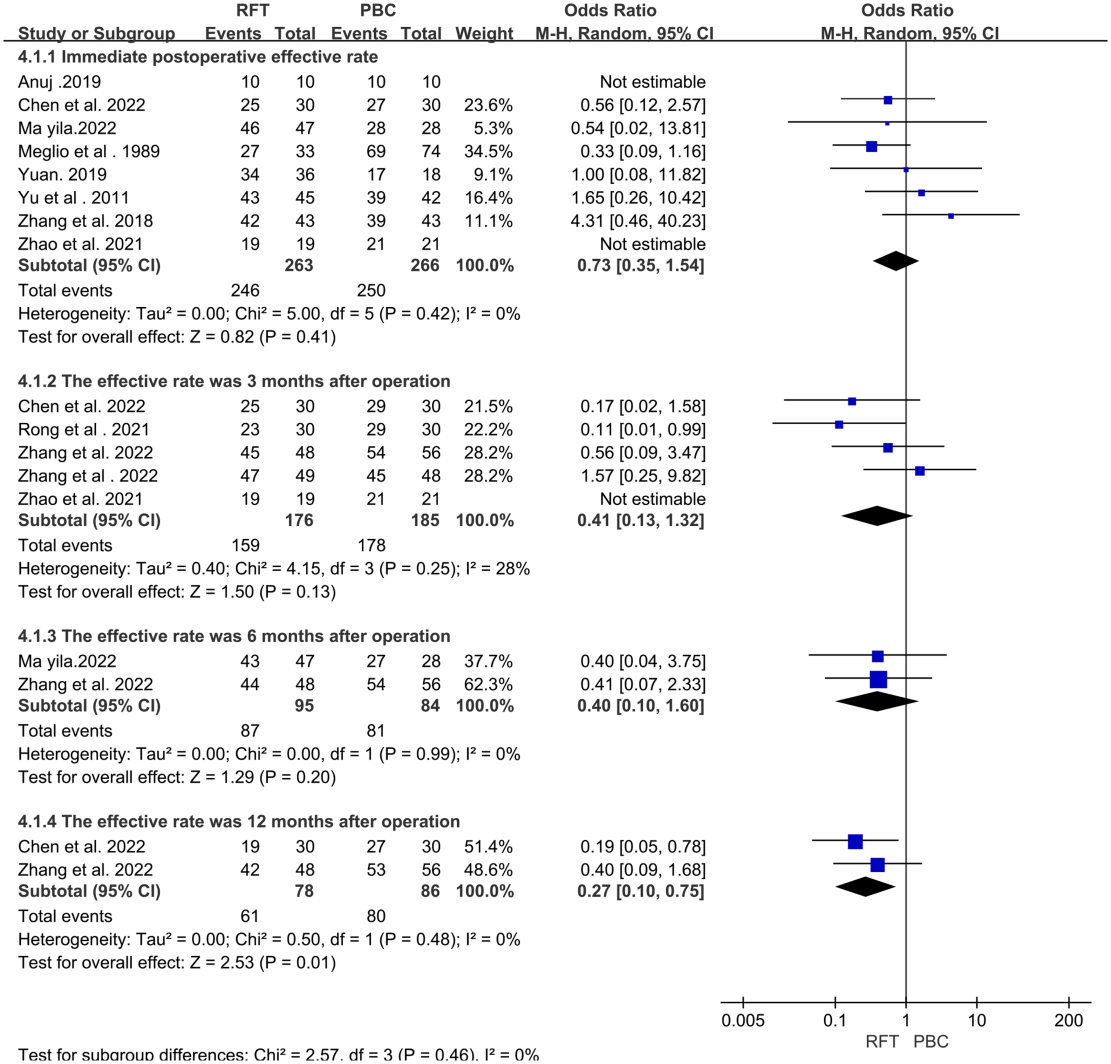
The effective rates were compared between the two groups immediately, 3 months, 6 months and 12 months after surgery.

An effective rate of 790 cases was reported in 11 articles (12–14, 17–21, 24). Heterogeneity analysis revealed that the included studies did not have good homogeneity. Further, a random effect model was used and subgroup analysis was performed (for immediate postoperative effective rates at 3, 6, and 12 months after surgery). No significant differences were observed in the effective rate between the two groups immediately (OR = 0.73, 95% CI 0.35–1.54, *p* = 0.41), 1 month (OR = 0.41, 95% CI 0.13–1.32, *p* = 0.13), and 3 months (OR = 0.40, 95% CI 0.10–1.60, *p* = 0.20) after surgery; however, 12 months after surgery, the difference was statistically significant (OR = 0.27, 95% CI 0.10–0.75, *p* = 0.01) (Figure 2).

### 3.3. Comparison of recurrence rates

**Figure 3:**
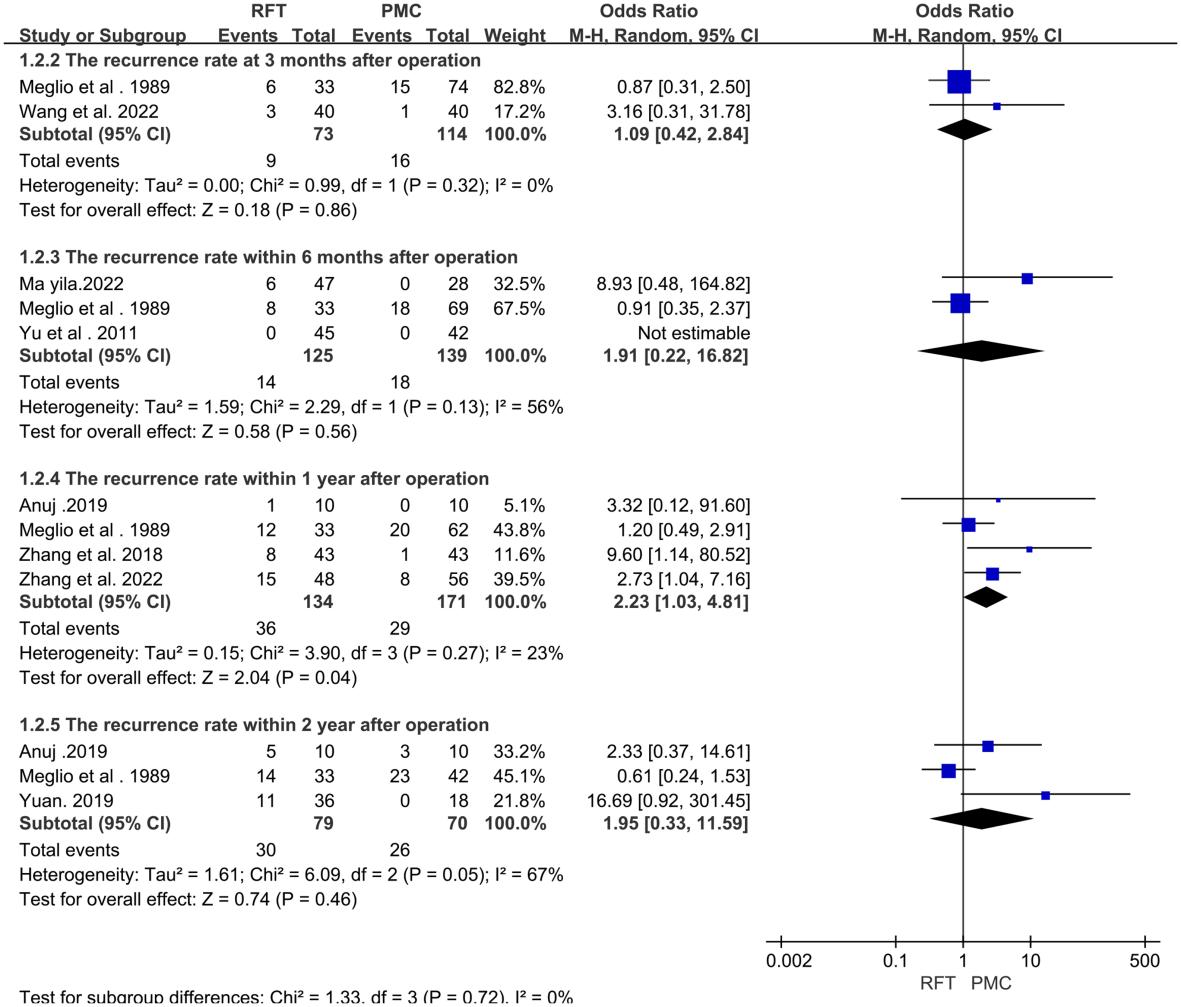
The recurrence rates of the two groups at 3 months, 6 months, 1 year and 2 years after surgery were compared.

In total, 613 cases of pain recurrence at discharge were reported in 8 articles (10, 11, 13–15, 18, 21, 24). Heterogeneity analysis revealed that the included studies did not have good homogeneity. Subgroup analysis was performed (for recurrence rates within 3 months, 6 months, 1 year, and 2 years after surgery) using a random effect model. A statistically significant difference was found in the recurrence rate between the two groups at 1-year follow-up after surgery (OR = 2.23, 95% CI 1.03–4.81, *p* = 0.04); however, no statistically significant differences were observed in the recurrence rate at 3-month (OR = 1.09, 95% CI 0.42–2.84, *p* = 0.32), 6-month (OR = 1.91, 95% CI 0.22–16.82, *p* = 0.13), and 2-year follow-ups after surgery (OR = 1.95, 95% CI 0.33–11.59, *p* = 0.46) (Figure 3).

### 3.4. Comparison of complication rates

**Figure 4:**
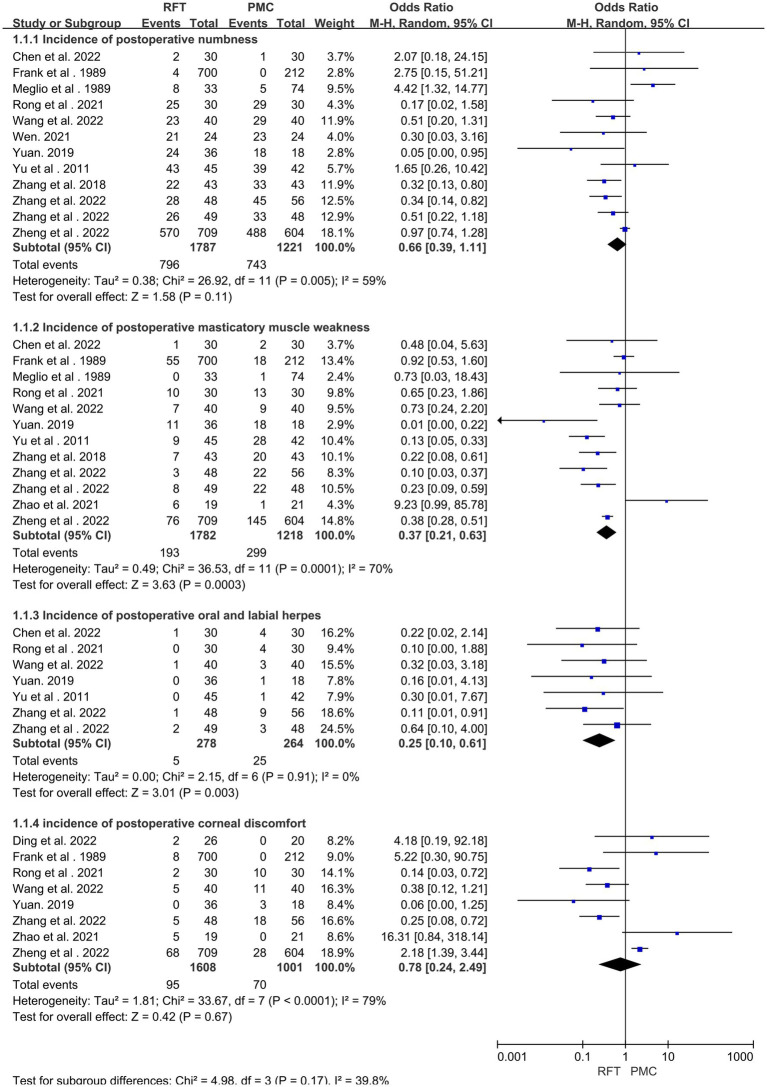
The incidence of complications was compared between the two groups.

Postoperative complications were reported in 14 articles (12, 14–23, 25) with 3,231 cases. Heterogeneity analysis revealed that the included studies did not have good homogeneity. A random effect model was used and subgroup analysis was performed (for facial numbness, masticatory muscle weakness, oral and labial herpes, and corneal discomfort). There were statistically significant differences in the incidence of decreased masticatory muscle strength and oral herpes postoperatively. However, no significant difference was found in the incidence of postoperative facial numbness (OR = 0.66; 95% CI 0.0.39–1.11, *p* = 0.11) and corneal discomfort (OR = 0.78; 95% CI 0.24–2.49, *p* = 0.67) (Figure 4).

### 3.5. Comparison of postoperative pain scores (NRS)

**Figure 5:**
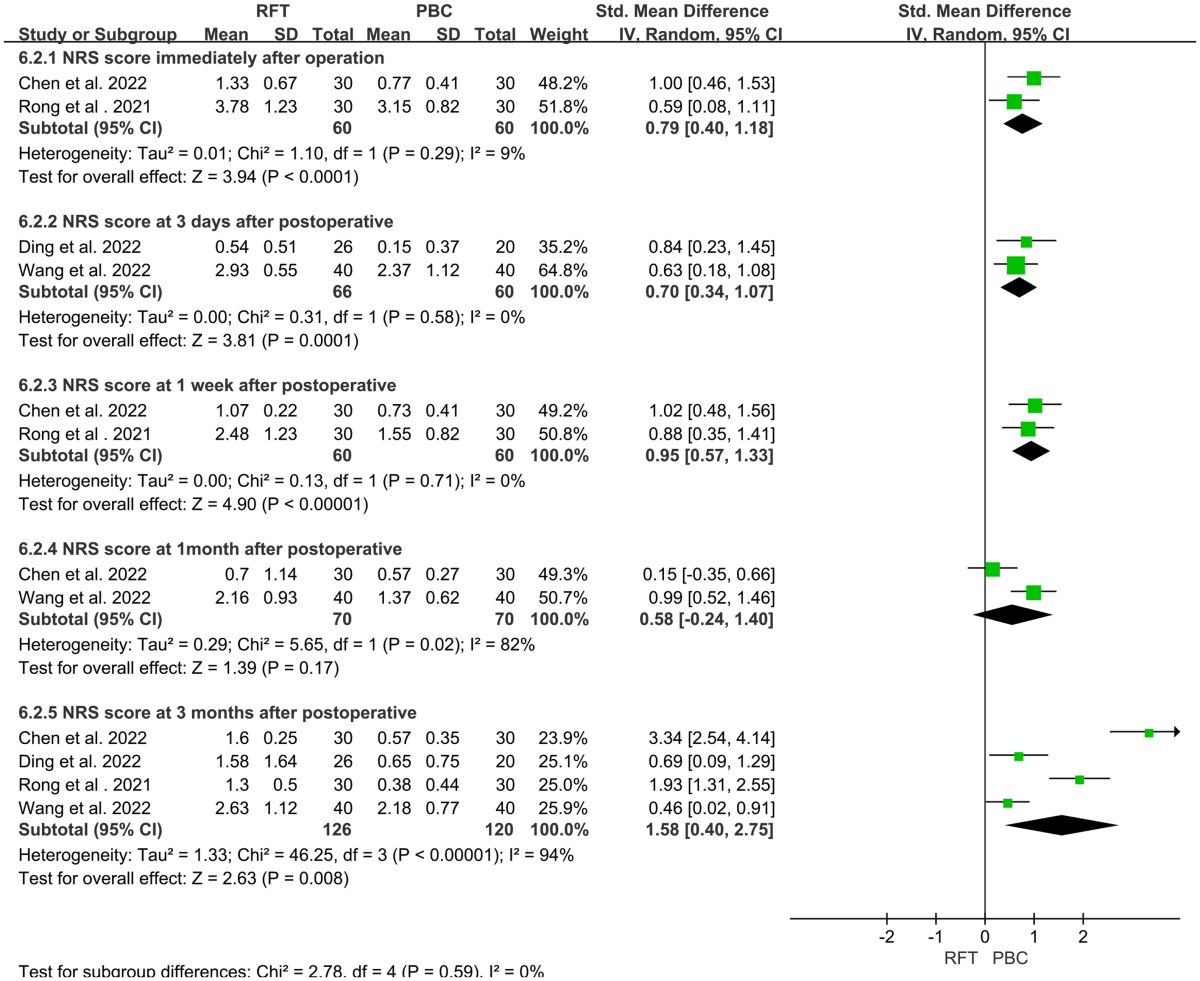
Comparison of postoperative pain scores (NRS) in the two groups.

Four articles (12, 15, 16, 19) reported 246 cases of postoperative NRS. Heterogeneity analysis indicated that the included studies did not have good homogeneity. Subgroup analysis was performed (for NRS scores immediately, 3 days, 1 week, 1 month, and 3 months after surgery) using a random effect model. The scores of the two groups were significantly different immediately (*SMD* = 0.79, 95% CI 0.40–1.18, *p* < 0.0001), 3 days (*SMD* = 0.70, 95% CI 0.34–1.07, *p* = 0.0001), 1 week (*SMD* = 0.95, 95% CI 0.77–1.33, *p* < 0.0001), and 3 months (*SMD* = 1.58, 95% CI 0.40–2.75, *p* = 0.008) after surgery. Notably, no statistically significant difference was found in the NRS score at 1 month after surgery (*SMD* = 0.58, 95% CI −0.24–1.40, *p* = 0.17) (Figure 5).

### 3.6. Comparison of postoperative pain scores (VAS)

**Figure 6:**
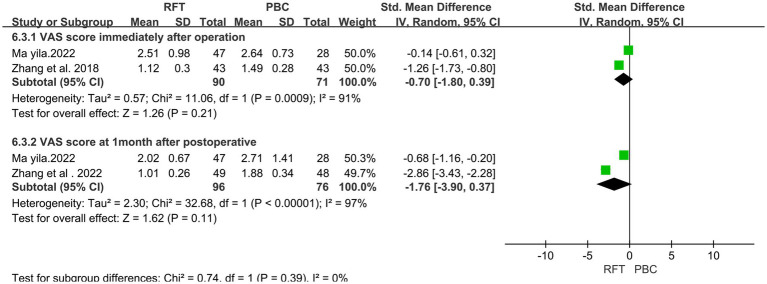
Comparison of postoperative pain scores (VAS) in the two groups.

In total, 3 articles (10, 13, 17) on postoperative VAS reported 258 cases. Heterogeneity analysis revealed that the included studies did not have good homogeneity. A random effect model was used, and the results of subgroup analysis (for VAS scores immediately and 1 month after surgery) indicated no significant differences in the VAS scores immediately (*SMD* = −0.70, 95% CI −1.80 to 0.39, *p* = 0.21) and 1 month (*SMD* = −1.76, 95% CI −3.90 to 0.37, *p* = 0.11) after surgery (Figure 6).

### 3.7. Score for postoperative sleep quality index (PSQI)

**Figure 7:**
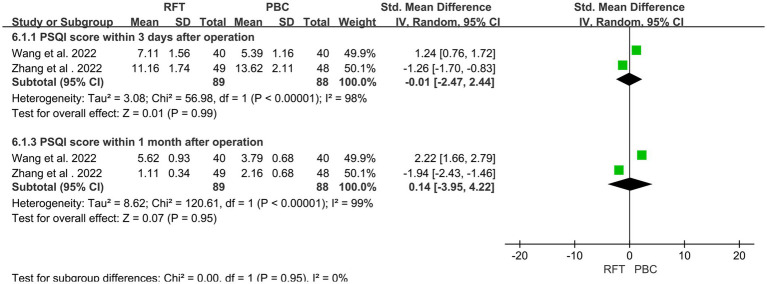
The postoperative sleep quality index (PSQI) scores of the two groups were compared.

Two articles (15, 17) on PSQI reported 177 postoperative cases. Heterogeneity analysis revealed that the included studies did not have good homogeneity. Subgroup analysis (for PSQI scores 3 days [*SMD* = −0.01, 95% CI 2.47–2.44, *p* = 0.99] and 1 month [*SMD* = 0.14, 95% CI 3.95–4.22, *p* = 0.95] after surgery) revealed no statistically significant differences between the two groups (Figure 7).

### 3.8. Sensitivity analysis

**Figure 8:**
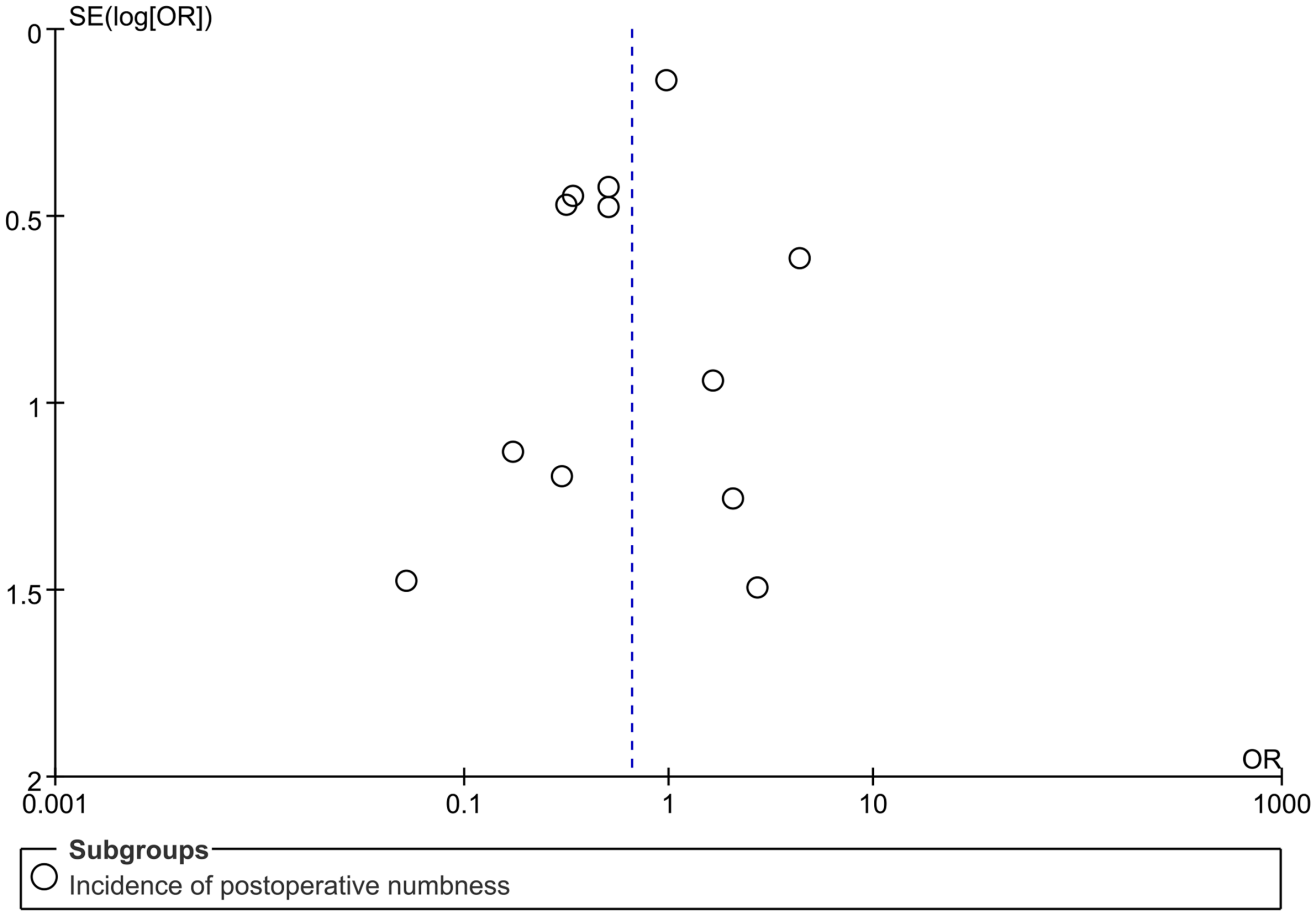
Study publication bias analysis.

A funnel plot was created using the postoperative numbness index. The plot was found to be symmetrical, indicating that the likelihood of publication bias was low (Figure 8).

## 4. Discussion

Recently, the treatment options for TN have become increasingly diverse. In addition, there are no uniform selection criteria for the treatment indications for TM, and the postoperative effective, complication, and recurrence rates vary greatly. Zheng (26) introduced two common surgical methods: (1) microvascular decompression using Teflon spacers to isolate the responsible blood vessels and (2) full release and decompression of the trigeminal nerve to achieve the therapeutic purpose. Notably, full release and decompression of the trigeminal nerve has the characteristics of a definite curative effect and a low recurrence rate. Conversely, microvascular decompression should be performed under general anesthesia, which has stringent requirements for both patients and operators and is not the best option for all patients with TN. According to Guo (27), stereotactic radiotherapy is an effective and minimally invasive procedure for the treatment of TN, with accurate position, good safety and effectiveness, and a few side effects. However, this method does not provide immediate pain relief and needs to be performed again after surgery, which itself has some limitations. Notably, RFT and PBC have rapid effect, little trauma, high safety factor, high pain relief rate, and few postoperative complications, and these methods are being performed by an increasing number of hospitals.

Radiofrequency thermocoagulation is used to achieve pain relief by destroying the fine fibers that conduct the sensation of facial pain in the semilunar ganglion and preserving the coarse fibers that conduct the sensation of touch and movement, which are more resistant to heat (28, 29). This procedure is often used in patients who cannot undergo craniotomy. Numbness of the face is the most common complication of RFT, but when heat coagulation is applied to the first branch of the trigeminal nerve, the end of the ophthalmic branch may be damaged, leading to corneal ulcers. Deng et al. (30) examined 70 patients with pain in the ophthalmic branch of trigeminal nerve and found that compared with RFT alone, RFT used in combination with pulse radiofrequency for corneal ulcers (17.1%, 6/35) is associated with a lower rate of complications (40.0%, 14/35). Because RFT is only performed under local anesthesia, it is highly selective for some patients with TN who cannot tolerate anesthesia. Notably, mechanical compression damages the thick myelinated nerve fibers while leaving the small myelinated nerve fibers and unmyelinated nerve fibers unaffected. To achieve the goal of pain relief, PMC can be used to block the pain conduction pathway and relieve the possible local compression of the trigeminal nerves Gasseric ganglion (31). Du et al. (32) reported that the balloon shape was an independent prognostic factor affecting the immediate disappearance of postoperative pain in 200 patients with TN following PBC treatment, and the location of the balloon should be adjusted to the maximum possible appearance of the pear shape. However, notably, a previous history of surgery may cause local adhesion of Meckel’s cavity, making it impossible to achieve the ideal pear shape, thereby affecting the surgical effect. Furthermore, Yu et al. (33) reported that the effect of PBC surgery was influenced by the balloon compression time as well as whether the balloon was pear-shaped. He Ruelin et al. (34) examined the short- and long-term effects and complications of different compression durations on patients with TN, and they found that the compression time of 120 s ensured effective treatment as well as reduced the incidence of facial numbness and masseter muscle asthenia. Furthermore, PBC may selectively damage pain-sensing nerve fibers while retaining unmyelinated nerve fibers that mediate the corneal reflex. In addition, PBC provides some benefits for the pain in the first branch of the trigeminal nerve.

Because the efficacy of the two surgical methods is controversial, therefore, this meta-analysis was conducted to discuss this situation. Eleven articles compared the effectiveness of the two surgical methods at various time points, including immediately after surgery and 3, 6, and 12 months after surgery. The results revealed no significant differences in the effective rate between the two groups immediately (OR = 0.73, 95% CI 0.35–1.54, *p* = 0.41), 1 month (OR = 0.41, 95% CI 0.13–1.32, *p* = 0.13), and 3 months (OR = 0.40, 95% CI 0.10–1.60, *p* = 0.20) after surgery; however, at 12 months after surgery, the difference was statistically significant (OR = 0.27, 95% CI 0.10–0.75, *p* = 0.01). In the short term (<12 months), no significant difference was found in the effective rate between the two groups. Both of these procedures have high efficiency after surgery. It is considered to achieve the purpose of analgesia by destroying the trigeminal nerve and destroying the pain perception conduction fibers. However, after ≥12-months, the effective rate of PBC was higher than that of RFT, It can be speculated that the long-term efficacy of PBC is superior than RFT, consistent with most studies (20, 35). The two surgical methods of the treatment of TN (i.e., PBC and RFT) are associated with different degrees of recurrence at follow-up; moreover, the recurrence rate is one of the reasons why patients choose surgery. Notably, the recurrence rate obtained by comparing eight articles and the results of the heterogeneity test revealed that the included studies did not have good homogeneity. The random effect model was used, and subgroup analysis was performed (for recurrence rates within 3 months, 6 months, 1 year, and 2 years after surgery). The results revealed a significant difference in the recurrence rate between the two groups at 1 year after surgery (OR = 2.23, 95% CI 1.03–4.81, *p* = 0.04), whereas no significant difference was noted in the recurrence rate at 3 months (OR = 1.09, 95% CI 0.42–2.84, *p* = 0.32), 6 months (OR = 1.91, 95% CI 0.22–16.82, *p* = 0.13), and 2 years (OR = 1.95, 95% CI 0.33–11.59, *p* = 0.46) after surgery between the two groups. Considering that the sample size of the two groups was small and there was certain heterogeneity in the 2-year comparison, the possibility of pain recurrence in the long term was lower in the group that underwent PBC than in the group that underwent RFT. This may be attributed to the fact that RFT causes nerve fiber damage, which may be incomplete, thereby inducing partial nerve cell regeneration and leading to a high long-term recurrence rate. Conversely, mechanical compression of PBC causes cell apoptosis, resulting in a low recurrence rate (15). Li et al. (36) used RFT to treat 438 patients with TN and followed up the curative effect of this treatment. They found that the causes of pain recurrence may be atypical pain, long course of disease, previous history of microvascular decompression or gamma knife treatment, and Barrow Neurological Institute Pain Intensity Score of grade V (severe pain that could not be controlled by medication). Shen et al. (37) reported that differences in sensitivity of patients to intraoperative balloon filling size and compression time may be related to the cause of postoperative pain recurrence following PBC.

In patients with TN, postoperative complications, such as facial numbness and decreased masticatory muscle strength, are common. The incidence of numbness following RFT was higher in four articles (11, 12, 14, 22), whereas the incidence of numbness following PBC was higher in seven studies (10, 15, 17–19, 21, 25); therefore, this meta-analysis was performed to further verify the results. Subgroup analysis was performed for 14 articles that reported complications (in terms of the incidence of postoperative numbness, decreased masticatory muscle strength, the occurrence of oral and labial herpes, and corneal discomfort). Statistically significant differences were observed in the incidence of decreased masticatory muscle strength (OR = 0.37, 95% CI 0.21–0.63, *p* = 0.0003) and oral and labial herpes (OR = 0.25, 95% CI 0.10–0.61, *p* = 0.003). However, no statistically significant difference was noted between the two groups in terms of postoperative numbness (OR = 0.66, 95% CI 0.0.39–1.11, *p* = 0.11) and corneal discomfort (OR = 0.78, 95% CI 0.24–2.49, *p* = 0.67). This could be attributed to the fact that the sensory nerve fibers are damaged in a short period in both methods, thereby resulting in a similar incidence of facial numbness. Owing to of the eye branch of the trigeminal nerve leaning up, PRF causes less direct damage to this branch, whereas PBC mainly mechanically compresses conduction nociception of large myelinated nerve fibers, does not damage myelinated or unmyelinated nerve fibers, thereby reducing the risk of postoperative ocular complications (38, 39). Thus, no statistically significant difference was noted in the prevalence of corneal discomfort. This study found that compared with RFT, PBC is associated with a higher incidence of decreased masticatory muscle strength and orolabial herpes. As PRT destroys nerve fibers while retaining motor nerve fibers, the mechanical compression of PBC may damage motor nerves to varying degrees, thereby increasing the rate of decreased masticatory muscle strength. Herpes simplex is closely related to the compression and activation of the herpes virus that lurks in the semilunar node of the trigeminal nerve. The most common complication in both groups was postoperative numbness (RFT: 796/1,787 patients; PBC: 743/1,221 patients). Wang et al. (40–42) reported that the main complications of RFT were moderate facial hypesthesia, pain sensory loss, masticatory muscle weakness, facial swelling, corneal ulcer, and abducens nerve palsy. Excessive radiofrequency temperature, incorrect positioning, multiple punctures, and punctures to the peripheral cranial nerves can cause various complications, and most of these complications resolve over time. Among them, the too high temperature is the main reason, studies have shown that, Inappropriately elevated temperature during RFT not only impairs Aδ and C unmyelinated nerve fibers, And also damage the Aα and Aβ fibers in a non-selective manner, Thus inducing serious complications, As in the Severe facial numbness, permanent masticatory atonia, and corneal hypoesthesia (43), For example, diplopia, hearing loss and ptosis may occur at 85°C (44), and abducens nerve damage and visual loss at 95°C (45). Therefore, currently recommended low-temperature RFT (60–75°C) for treatment of TN (46). In the study by Wang Bin et al. (47), the most common complications in the PBC treatment of 1,263 patients with TN were hypesthesia with paresthesia, ipsilateral masticatory muscle weakness, and keratitis. Further, Li et al. (48) reported that the complications can be caused by an incorrect balloon position, insufficient balloon compression time and filling pressure, simultaneous compression of the balloon to the trigeminal ganglion and motor branch, deviation of the needle from the planned puncture direction, and anatomical variation of the skull base. In addition to the compression time, the shape of the balloon is also important, Until now, a pear-shaped balloon may have been the only factor influencing the outcome of PBC in TN treatment (34, 49, 50).

NRS and VAS are quantitative indicators used to assess postoperative pain among patients. Three studies have reported postoperative VAS scores, and subgroup analysis was performed. No significant difference was found in the VAS scores between the two groups immediately and/or 1 month after surgery. The NRS scores at discharge were reported in four articles, and subgroup analysis was performed. The NRS scores of the two groups were significantly different immediately after surgery and 3 days, 1 week, and 3 months after surgery; however, no significant difference was noted at 1 month after surgery. PSQI is a sleep quality score that indicates pain relief among patients. Subgroup analysis of two articles that reported PSQI at discharge was performed. No significant differences were observed in the pain and sleep scores within 1 month after surgery, whereas the pain score of the PBC group was lower than that of the RFT group 3 months after surgery, indicating that the medium- and long-term efficacy of PBC may be better than that of RFT. Both procedures are believed to produce analgesia by destroying the structure of nerve fibers, and their efficacy is dependent on the degree of nerve fiber destruction. PBC mechanical compression following balloon filling causes more direct damage, with a more definite long-term effect, Part of the nerve fibers after RF were regenerated, so the pain occurred again, and the pain score gradually increased.

There is no definite answer to the question of which type of treatment is the most effective. To the best of our knowledge, this is the first meta-analysis comparing balloon compression with RFT for TN treatment. However, this study has some limitations. First, because the number of studies was limited, errors associated with the chance were unavoidable and may have introduced bias in the results. Second, the meta-analysis included some low-quality and small-sample studies, and these studies were mostly retrospective in nature, which may have introduced bias in the results. Third, the RFT radiofrequency time, thermal coagulation temperature, and puncture method were not entirely consistent across all patient groups in the present study. Moreover, the balloon compression time and filling pressure in PBC were different, and the evaluation criteria were not the same; therefore, the results of this analysis may be affected. Finally, we must objectively assess heterogeneity and publication bias; therefore, larger well-designed randomized controlled trials are needed to confirm our findings.

## 5. Conclusion

The results of the present study indicated that RFT and PBC have good medium-term efficacy in the treatment of TN, with no significant differences in pain relief rate, comprehensive pain score, or recurrence rate between the two groups within 3 months after surgery. PBC may have a lower pain score and recurrence rate than RFT in the long term. Conversely, RFT is associated with fewer postoperative complications, including decreased masticatory muscle strength and oral and labial herpes. Finally, after completely evaluating the indications and contraindications, appropriate treatment can be selected for patients with TN who have poor drug effects and cannot tolerate craniotomy based on the advantages and disadvantages of the two surgical methods.

## Author contributions

YF designed the study. ZW, YZ, and YY selected the articles and retrieved the data. ZW and YF collected and analyzed the data and drafted the manuscript. JL provided revision suggestions and language help during the revision of the manuscript. All the authors approved the final version of the manuscript.

## Funding

This study was supported by Sichuan Provincial Administration of Traditional Chinese Medicine Fund (2021MS352).

## Conflict of interest

The authors declare that the research was conducted in the absence of any commercial or financial relationships that could be construed as a potential conflict of interest.

## Publisher’s note

All claims expressed in this article are solely those of the authors and do not necessarily represent those of their affiliated organizations, or those of the publisher, the editors and the reviewers. Any product that may be evaluated in this article, or claim that may be made by its manufacturer, is not guaranteed or endorsed by the publisher.
